# On the Duration and Extent of the Protective Power of Vaccination

**Published:** 1852-12

**Authors:** O. C. Gibbs

**Affiliations:** Perry, Lake County, Ohio


From the American Medical Journal.

*On the Duration and Extent of the Protective Power of Vaccination, by* O. C. GIBBS, M. D., of Perry, Lake County, Ohio.

The question concerning the duration and extent of the protective power of vaccination, has from time to time, since the days of the illustrious Jenner, engaged the serious attention of the medical profession. As yet, the anxious question remains unsettled. Dr. Gregory, of London, as well as others, have of late been trying to shake that confidence which the sanguine predictions of Jenner inspired. Among the conflicting opinions, the public are anxious to know which is the right; are anxious to know what is the *extent* and *duration* of the protective power of vaccination.

Dr. Gregory asserts that the protecting power of vaccination is far less than of variolous inoculation; and also, that the comparative slight safety which it offers is but *temporary* in duration.

It would seem as though the combined experience of the medical profession might set this question forever at rest, if that experience was based upon unexceptionable observation. My experience, like that of every country practitioner, who has numbered but a few years of practical observation, is slight, yet such as it is, it bears directly upon the question under notice.

In the winter of 1850, I was called to see a boy who had never been vaccinated, and in whose case the variolous eruption was just making its appearance. There were six other members in the family, varying from two to forty years of age, all of whom had been vaccinated but the two youngest, whose ages were respectively, two and four years. The parents had been vaccinated when young; I had been vaccinated when but eight years of age, and neither they nor I had been revaccinated, neither were we. On the following day, I vaccinated those who had not previously been, with vaccine matter which was several years old. The vaccine disease was produced in the oldest of the two, but not in the young one. On the third day from the first vaccination, I revaccinated the younger, and the vaccine disease failing to appear, on the third day from the last vaccination I performed variolous inoculation; but, fortunately, on the following day, the vaccine disease was manifested through the first vaccination, and it run its regular course, rendering the variolous inoculation abortive. This child, the rest of the family, and myself, all escaped the disease. There was but one room in the house, and that confined and filthy.

The mother was four months pregnant, went her full time, and was delivered of a child presenting no marks of previous disease.

Since the above, I have attended one case in a family of five persons, all of whom had been vaccinated but the subject of the disease. The remainder of the family escaped the small-pox, though the parents had been vaccinated for at least thirty years, and had never been revaccinated.

Here are persons repeatedly exposed with impunity to the variolous contagion, who have had the vaccine disease thirty years before, and others, with equal impunity, who took the cow-pox eight days after the small-pox exposure.

These facts, of course, are too limited from which to draw a safe conclusion; yet, they induce us to hope that an accumulation of closely scrutinized observations may lead us to a more favorable conclusion than that advanced by Dr. Gregory and his compeers in opinion.

Perry, Lake county, Ohio, August 20, 1852.

